# ID 54 - Ecossistema para Fortalecimento da Avaliação de Tecnologias em Saúde nas Secretarias Estaduais de Saúde: uma ferramenta estratégica

**DOI:** 10.5327/2237-9622.2025.v34s1.54

**Published:** 2025-11-25

**Authors:** Rodrigo Pereira de Almeida, Bruna Marmett, Ana Paula Blankenheim, Bárbara Cristiane da Silva, Roseana Boek Carvalho, Marina Petrasi Guahnon, Muriel Primon de Barros, Gilson Pires Dorneles, Maicon Falavigna, Suena Medeiros Parahiba

## Abstract

**Introdução:**

A iniciativa envolve o desenvolvimento de um ecossistema integrado, como ferramenta de disseminação de Avaliação de Tecnologias em Saúde (ATS), destinado aos profissionais das Secretarias Estaduais de Saúde (SES). Por meio da utilização de diferentes abordagens, a fim de maximizar o alcance e a interação com o público-alvo, a ferramenta foi desenvolvida para facilitar o acesso à informação de forma dinâmica, promovendo assim a capacitação contínua e discussão ativa entre os profissionais. O ecossistema foi estruturado em quatro principais pilares:

– Informação: A disseminação de informações sobre ATS é feita por meio de um boletim informativo mensal, que apresenta de forma acessível e objetiva conceitos e atualizações no campo da ATS, facilitando o rápido compartilhamento de conhecimento entre os profissionais.
– Comunicação: A criação de uma rede de comunicação entre os profissionais foi realizada por um grupo de WhatsApp. Esse ambiente de comunicação rápida foi destinado à troca de informações, documentos e divulgação de eventos na área de ATS, promovendo um canal acessível e funcional.
– Discussão: O ambiente de discussão se dá por meio de webinários mensais. Estes encontros, on-line e síncronos, foram estruturados para apresentar temas relacionados à ATS, incluindo convidados das próprias SES para expor suas experiências na área.
– Capacitação: A capacitação dos profissionais é feita por uma trilha de cursos gratuitos, autoinstrucionais e on-line, que abordam desde temas introdutórios como síntese da evidência até temas mais avançados, como avaliação econômica e avaliação da certeza da evidência. Essa trilha está disponível a todos os profissionais e permanecerá acessível até o final de 2026.

A iniciativa está alinhada com o objetivo de promover a disseminação de ações de ATS por meio de diferentes formatos e mídias, proporcionando uma linguagem acessível e interativa que consiga atingir diferentes públicos. A apresentação do ecossistema será feita por meio da exposição de cada um dos pilares, seus respectivos formatos e os principais resultados, com apoio de uma apresentação visual.

**Método:**

Durante os 5 minutos de apresentação, abordaremos os seguintes tópicos:

– Introdução à iniciativa (1 minuto): Visão geral do ecossistema de disseminação de ATS das SES, destacando sua importância e impacto nas ações de saúde pública.
– Exposição da abordagem de cada pilar do ecossistema (3 minutos):

**Informação:**

Apresentação da estrutura principal dos documentos e das principais pautas abordadas, trazendo um dos boletins como exemplo.

**Comunicação:**

Demonstração das trocas de experiências entre os profissionais, divulgação de eventos e compartilhamento de materiais no grupo de mensagens.

**Discussão:**

Apresentação dos dados de participação dos profissionais das SES nos webnários, bem como dos temas discutidos.

**Capacitação:**

Apresentação dos dez cursos da trilha de conhecimento, destacando os temas, carga horária e outras informações relevantes.

**– Resultados e impacto (1 minuto):**

Breve exposição dos dados de alcance das ferramentas e do ecossistema, como número de participantes no grupo de WhatsApp, nos encontros mensais, profissionais inscritos na lista de transmissão dos boletins, e número de participantes nos cursos da trilha de conhecimento.

**Equipamento necessário:**

Projetor para exibição de eslaides com dados visuais e de cada uma das ferramentas.

